# History-Dependent Odor Processing in the Mouse Olfactory Bulb

**DOI:** 10.1523/JNEUROSCI.0755-17.2017

**Published:** 2017-12-06

**Authors:** Amit Vinograd, Yoav Livneh, Adi Mizrahi

**Affiliations:** ^1^Department of Neurobiology, Institute of Life Sciences, and; ^2^The Edmond and Lily Safra Center for Brain Sciences, The Hebrew University of Jerusalem, Edmond J. Safra Campus, Givat Ram, Jerusalem 91904, Israel

**Keywords:** olfactory bulb, olfactory coding, two-photon calcium imaging

## Abstract

In nature, animals normally perceive sensory information on top of backgrounds. Thus, the neural substrate to perceive under background conditions is inherent in all sensory systems. Where and how sensory systems process backgrounds is not fully understood. In olfaction, just a few studies have addressed the issue of odor coding on top of continuous odorous backgrounds. Here, we tested how background odors are encoded by mitral cells (MCs) in the olfactory bulb (OB) of male mice. Using *in vivo* two-photon calcium imaging, we studied how MCs responded to odors in isolation versus their responses to the same odors on top of continuous backgrounds. We show that MCs adapt to continuous odor presentation and that mixture responses are different when preceded by background. In a subset of odor combinations, this history-dependent processing was useful in helping to identify target odors over background. Other odorous backgrounds were highly dominant such that target odors were completely masked by their presence. Our data are consistent in both low and high odor concentrations and in anesthetized and awake mice. Thus, odor processing in the OB is strongly influenced by the recent history of activity, which could have a powerful impact on how odors are perceived.

**SIGNIFICANCE STATEMENT** We examined a basic feature of sensory processing in the olfactory bulb. Specifically, we measured how mitral cells adapt to continuous background odors and how target odors are encoded on top of such background. Our results show clear differences in odor coding based on the immediate history of the stimulus. Our results support the argument that odor coding in the olfactory bulb depends on the recent history of the sensory environment.

## Introduction

Natural environments comprise complex stimuli, which are actively processed by the nervous system to extract behaviorally relevant information. Moreover, at any given time point, animals face sensory environments that were immediately preceded by other environments. The moments preceding a sensory stimulus may contain vital information for the animal or simply be noise that should be suppressed. Behaviorally, animals often can seamlessly deal with background and perceive details in noisy and dynamic scenes. In other cases, background can be so dominant that it makes the perception of novel odors impossible.

Backgrounds have been shown to alter processing in various sensory systems. For example, in the retina, neurons increase their sensitivity in response to visual patterns differentially oriented from their background (Hosoya et al., 2005). In the visual cortex of cats, adaptation was shown to be pattern selective (Movshon and Lennie, 1979). In the primary auditory cortex of mice, responses are highly sensitive to the background with which they are embedded in time and frequency (Williamson et al., 2016). Similarly, in the barrel cortex of rats, high sensitivity across whiskers is maintained following an adaptive response to high-frequency whisker stimulation (Katz et al., 2006). In olfaction, neural correlates of background processing have been studied in only a few cases, so our understanding of these processes remain rudimentary.

Olfactory backgrounds are often continuous and dynamic. As such, they must be processed on both fast and slow time scales. Although significant computations do occur at short time scales, the effects of slowly changing or continuous stimuli on odor coding are normally not addressed (Cleland, 2010; Carandini and Heeger, 2011; Koulakov and Rinberg, 2011; Friedrich and Wiechert, 2014). Moreover, empirical data on the extent to which slow odor responses change and how these contribute to coding are not only scarce but conflicting. In some studies, background odor information passing through the olfactory bulb (OB) was reported to remain unperturbed (Kadohisa and Wilson, 2006; Stevenson and Wilson, 2007; Gottfried, 2010; Tong et al., 2014), while in other studies it was shown to be dynamically changing (Bathellier et al., 2008; Adam and Mizrahi, 2011; Patterson et al., 2013). Prevailing ideas suggest that neural correlates of odor coding over background in the mammalian olfactory system start to appear only in the piriform cortex, where strong adaptation to constant background odors has been measured (Gottfried, 2010; Wilson and Sullivan, 2011).

Here we studied odor coding in the presence of continuous background in the mouse OB using two-photon calcium imaging. We asked how mitral cells (MCs) process background odors and, in turn, how background odors affect transient odor-evoked responses by MCs. Our results suggest that odor coding is history dependent because odor responses to the same mixture stimulus changes when preceded by background. Moreover, history-dependent coding in the OB can either promote an effective detection of target odors or effectively suppress responses.

## Materials and Methods

### 

#### 

##### Animals.

We used Thy1-GCaMP3 (Chen et al., 2012) male mice (8–14 weeks old). Animal care and experiments were approved by the Hebrew University Animal Care and Use Committee.

##### Surgical procedures.

We anesthetized mice with an intraperitoneal injection of ketamine and medetomidine (100 and 0.83 mg/kg, respectively) and a subcutaneous injection of carprofen (0.004 mg/g). Additionally, we injected mice subcutaneously with dextrose–saline to prevent dehydration. We assessed the depth of anesthesia by monitoring the pinch-withdrawal reflex and added ketamine/medetomidine to maintain it. We continuously monitored the animal's rectal temperature and maintained it at 36 ± 0.5°C. For calcium imaging, we made a small incision in the animal's skin and glued a custom-made metal bar to the skull using dental cement to fix the head for imaging under the microscope. For acute imaging, we performed a craniotomy (2 × 1 mm) over the OB of one hemisphere. We placed 1.5% low-melting agar (type IIIa, Sigma-Aldrich) over the craniotomy covered by a glass cover, which was then secured with dental cement. For awake experiments, a single 3-mm-diameter craniotomy was opened over the OBs of both hemispheres using a 3 mm biopsy punch (Miltex), and the bone was carefully removed. The exposed brain was covered directly with a 3-mm-diameter round cover glass (Menzel-Glaser). The margin between the cover glass and the intact bone was gently sealed with Histoacryl glue (B. Braun). After surgery, mice were treated with carprofen (0.004 mg/g, s.c.) until full recovery. All animals were allowed to fully recover before the first imaging session, which started ≥2 weeks after surgery.

##### Two-photon calcium imaging.

We performed calcium imaging of the OB using an Ultima two-photon microscope from Prairie Technologies, equipped with a 16× water-immersion objective lens (0.8 numerical aperture; CF175, Nikon). We delivered two-photon excitation at 950 nm using a DeepSee femtosecond laser (Spectraphysics). The size of an imaging field was 169 × 169 μm (420 × 210 pixels). Acquisition rate was ∼7 Hz. Before awake imaging and 2 weeks after implanting the window, we habituated the mice under the microscope in the head-fixed position. Each mouse was habituated once a day for 15 min for 4 d. Awake imaging was performed in habituated mice, which showed no obvious signs of stress.

##### Odor delivery.

To deliver odorants, we used a custom-made olfactometer. To avoid cross-contamination between odorants, we used for each channel separate tubing from the odor vial to the animal's nose. We used a panel of seven odorants [ethyl-acetate (Ea), methyl-propionate (Mp), ethyl-tiglate (Et), butanal (Bu), ethyl-butyrate (Eb), isoamyl acetate (Ia), and propanal (Pr); all obtained from Sigma-Aldrich] and one blank channel with no odor inside. We presented odorants in a final concentration of 250 ppm at a rate flow of 1 l/min. The short stimuli lasted 2 s with a 15 s interstimulus interval. The target-over-background [t(B)] stimuli lasted 50 s with a 60 s interstimulus interval. We repeated all stimuli four times in a pseudorandom order. To equalize flow rates and concentrations between the different stimuli, we delivered each target odor at 500 ml/min together with a 500 ml/min blank stimulus and mixture stimuli at 500 ml/min for each odor. In the t(B) protocol, we delivered the background odor together with the blank (500 ml/min for each). When t(B) was presented, the blank stimulus was turned off and the target stimulus was turned on. Odor delivery was monitored using photoionization detector (miniPID, Aurora Scientific). In a separate set of experiments, we repeated the same protocol but with 10-fold lower odor concentration (25 ppm).

##### Data analysis.

We analyzed all data using custom-written code in Matlab (Mathworks). Regions of interest corresponding to individual cell bodies were manually drawn and the mean fluorescence of each cell body was extracted by ImageJ and exported to Matlab for analysis. Relative fluorescence change (Δ*f*/*f*) was calculated as follows: baseline fluorescence (*f*0) was the mean fluorescence over 2 s before odor onset. Traces were low-pass filtered using a square filter with a three-sample window. Zero-phase filtering was achieved by two passes of the filter using the Matlab filtfilt function. In awake imaging data, small lateral movements were corrected using cross-correlation image alignment. In a few cases (<1% of all responses) the responses were calculated based on three trials (instead of four). Coefficient of variance was calculated as the variance of peak amplitudes between each trial for all MCs divided by their mean. Decay times were calculated by an exponential fit from the peak amplitude to steady state using the “fit” function in Matlab.

Principal component analysis (PCA) was performed considering the entire recording period from the beginning of the odor stimulation to the end of recording for all different stimuli of all trials. To quantify distances between responses, we created response vectors from all cells measured in each individual mouse. These vectors were composed from the mean Δ*f*/*f* response values of the neurons at peak amplitudes for the solo and mixture odors (2 s after stimulus onset) and the same time after t(B) onset (42 s). Each vector was normalized to the number of MCs in each mouse, and the distance was calculated using the Euclidean distance between the vectors. Classifications were done using the Matlab function TreeBagger (200 trees). Each training stimulus was a vector composed of four trials at the peak amplitude of the stimulus (2 s after stimulus onset). Test trials were composed from the mean of four trials at the average time of peak amplitudes [2 s for single odors and mixtures, and 42 s for t(B)]. Shuffled data was created by shuffling the test stimuli across neurons for the same stimulus.

##### Experimental design and statistical analysis.

We recorded calcium transients in the OB of anesthetized and awake mice. Calcium transients were categorized as odor-evoked responses if all trials in addition to the mean had three consecutive Δ*f*/*f* values within the response window that were found to be above the mean + 1.6 SD of the values in the blank trial. Only cells that were responsive to ≥2 odors were included in the single-cell response analysis (see Figs. 3–5). We used an unpaired two-sample *t* test to compare the solo responses to the t(B) with the t(B)-baseline (nonparametric tests yielded similar results). All comparisons were done between the time bin of the averaged peak in the solo protocol and the equivalent time bin of the target stimulus in the t(B) protocol. Mixture changes were measured between the average peak amplitude of each component and the peak amplitude of their mixture. To calculate adaptive responses, we compared the maximum values of four trials at the beginning of the background stimulus (first 5 s) to the maximal values just before the target onset (35–40 s) using an unpaired two-sample *t* test. Responses significantly lower at 35–40 s were considered suppressed and classified as “adapting,” significantly higher trials were classified as “increasing” and those that were not significantly different were classified as “no change.”

## Results

**Figure 1.**
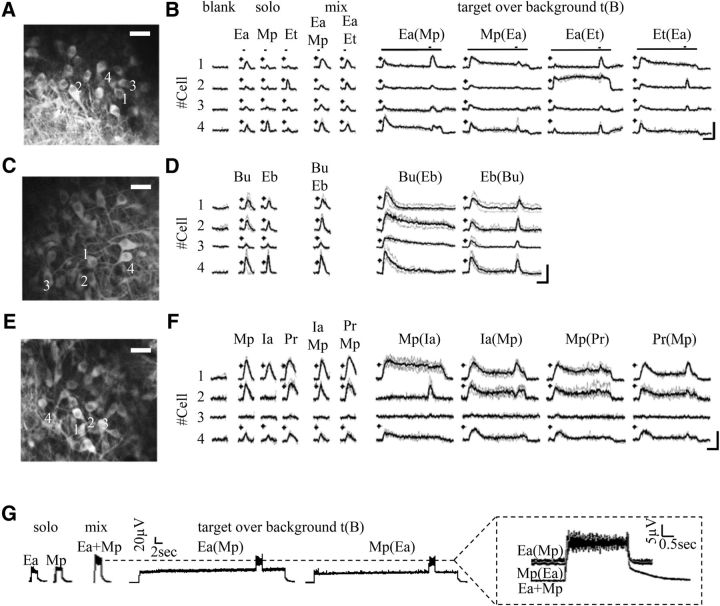
The experimental protocol. ***A***, *In vivo* two-photon micrograph showing a field of view of GCaMP3-expressing MCs at 267 μm below the dorsal surface of the main OB. Scale bar, 50 μm. ***B***, Representative examples of odor responses from four MCs in ***A***. The odor stimuli used are Ea, Mp, Et, and mix (binary mixtures). t(B) indicates the t(B) protocol where t is the target and B is the background. The target odor is delivered starting at 40 s after background onset. Asterisks indicate statistically significant responses. Scale bars: 50% Δ*f*/*f*; 10 s. ***C***, ***D–F***, Additional examples from different mice using different odors. Details the same as in ***A*** and ***B*** for the following odors: Bu, Eb, Ia, Pr. ***G***, Photoionization detector traces of the full protocol for two odors in isolation (Ea and Mp), their mixture (Ea+Mp), and the t(B) stimuli [Ea(Mp) and Mp(Ea)]. Inset, A zoom in on the traces comparing the mixture and the two t(B) conditions. Note that the mixture and the t(B) show identical traces and no deleterious effects of our olfactometer.

We used *in vivo* two-photon calcium imaging to test how MCs respond to a continuous odor as background, and how odor coding is affected by this background activity. We imaged the activity of MCs in Thy1-GCaMP3 mice expressing the genetically encoded calcium indicator GCaMP3 in MCs (Chen et al., 2012). MCs were identified morphologically by their large somata at ∼250–300 μm under the surface of the brain (Fig. 1*A*,*C*,*E*). To evaluate MC activity, we used seven monomolecular odors and their mixture pairs known to activate the dorsal surface of the OB: Ea, Mp, Et, Bu, Eb, Ia, and Pr (Adam et al., 2014; Livneh et al., 2014).

We focused on a protocol in which one odor becomes “background” due to its prolonged presence and another odor is considered “target” because it is transiently presented on top of the prolonged background odor (Kadohisa and Wilson, 2006; Gottfried, 2010; Saha et al., 2013). For each odor pair, the full protocol included five stimuli: two presented alone (diluted by air only and referred here as “solo” odors), their binary mixture, their t(B) combinations (where the odor indicated in parenthesis corresponds to the background), and a blank stimulus (Fig. 1*B*,*D*,*F*). We first tested responses to a continuous background odor alone. Then, we examined the responses to t(B) and compared these to responses of the target stimulus without background (solo).

The t(B) stimuli were composed of a 50 s “background,” and a 2 s “target” starting 40 s after the initiation of the background stimulus (Fig. 1*B*). A total of 10 odor pairs were examined in the t(B) protocol (Fig. 1*B*,*D*,*F*). In each imaging field, we collected data from a few dozen MCs simultaneously (average ± SEM: 34.9 ± 2.3 MCs). Representative examples showing a single field of imaging and responses from four MCs are shown in Figure 1*A*,*B* for the odors Ea, Mp, and Et. Additional examples from different fields and odors are shown in Figure 1*C–F*.

### Background suppression in the OB

We started by imaging anesthetized mice, where we collected responses from many MCs under stable physiological conditions. To increase the number of cells responding to ≥2 odors, we initially used a relatively high odor concentration (250 ppm) and collected data from 1325 MCs (*n* = 10 mice). Under these conditions, 1453 cell–odor pair responses to background were measured. Importantly, odor concentration during delivery remained largely stable across the 40 s stimulation of background odors (Fig. 1*G*).

**Figure 2.**
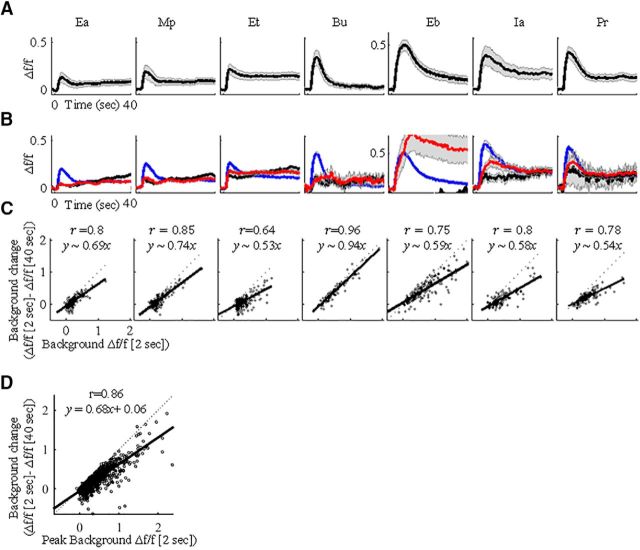
MCs adapt to background in an intensity-dependent manner. ***A***, MC responses to seven different background odors (duration, 40 s). Black, Mean traces; gray, SEM. ***B***, Same responses as in ***A*** only separated by classification: adapting, blue; no change, red; rising, black. ***C***, Linear correlations between background intensity at the peak (peak at 2 s, *x*-axis) and after background change (peak at 2 s − peak at 40 s, *y*-axis), per odor. Black curves reflect the linear regression equation on the top. Dotted line here and throughout the manuscript is when *x* = *y*. ***D***, Linear correlations between background intensity and background change, combined for all odors.

Calcium responses to background odor stimulation were frequently adaptive. The mean initial calcium response peaked at ∼2 s after stimulus onset and receded shortly thereafter (Fig. 2*A*). Fifteen seconds after stimulus onset, mean responses were already significantly lower, reaching ∼50% of their initial peak (0.34 ± 0.01 Δ*f*/*f* at the peak, 0.18 ± 0.01 at 15 s, *p* < 0.001, two-sided Wilcoxon signed-rank test). This decrease remained stable at 40 s after stimulus onset (which is just before the time when the target odor was presented; Fig. 2*A*; 0.16 ± 0.01 at 40 s). Despite the significant trend of the general adaptation, individual MC responses were heterogeneous (Fig. 2*B*). On average, 71.3% of the responses adapted per odor during the continuous background odor (Fig. 2*B*, blue), 18.3% remained unchanged after 40 s of background (Fig. 2*B*, red), and 10.4% showed a gradual increase (Fig. 2*B*, black). The adaptation of a response was positively correlated with the strength of the initial response to the background. Stronger initial responses were adapted strongly and weak responses showed little adaptation or even a gradual increase (Fig. 2*C*, each odor individually; *D*, all odors combined).

### MCs responses to t(B)

**Figure 3.**
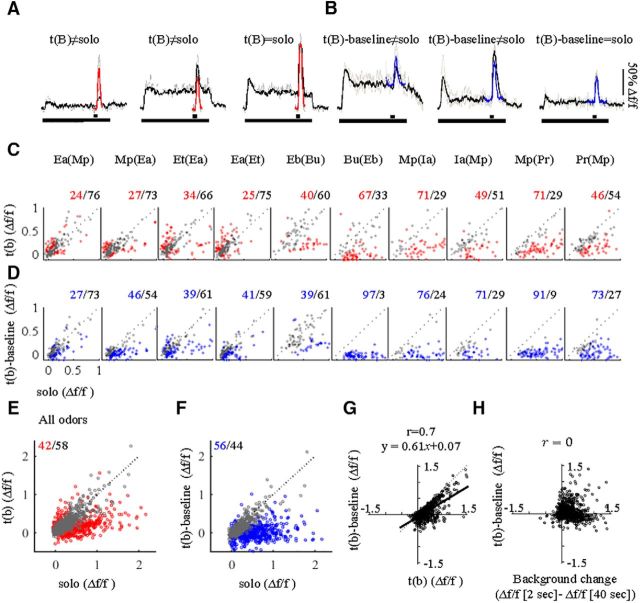
MCs responses to t(B). ***A***, Representative examples of calcium responses to the t(B) stimuli [black traces, drawn with reference to *f*(0)] compared with solo stimulus [red, solo response, drawn with reference to *f*(0)]. ***B***, Representative examples of calcium responses to t(B) stimuli [black traces, drawn with reference to *f*(0)] compared with the solo response (blue, drawn with reference to the background level before target onset). ***C***, Scatter plots for all odors separately comparing the maximum calcium response of the solo (*x*-axis) to its response as a t(B) (*y*-axis). Each data point is one cell–odor pair. Red dots and red numbers are responses where t(B)≠solo (unpaired 2-sample *t* test, *p* < 0.05), and gray dots and black numbers represent t(B)=solo. ***D***, Same as ***C*** but the t(B) response is calculated from background baseline rather than *f*(0) [t(B)-baseline; *y*-axis]. ***E***, ***F***, Same as ***C*** and ***D*** but for all odors combined. ***G***, Correlation between t(B) [when amplitude is measured from *f*(0); t(B), *x*-axis] and t(B)-baseline (when amplitude is measured from the baseline of background [t(B)-baseline, *y*-axis]. Black curve reflects the linear regression equation on the top. ***H***, t(B)-baseline responses (*y*-axis) versus the level of adaptation to the background odor before target onset (Background change, *x*-axis).

Using the same dataset described above (*n* = 1325), we next asked to what extent MC adaptation affects responses to additional odors when these odors appear on top of background. We refer to these new odors as t(B) or simply as target. To study how background affected individual MC responses, we analyzed MCs responding to both background and target odors. Out of the 1325 MCs recorded, 430 MCs responded to both background and target odors for a total of 1258 cell–odor pairs. Solo odors showed a range of response magnitudes (Figs. 1*B*,*D*,*F*, 3*A*,*B*, red and blue traces). t(B) responses were heterogeneous as well (Fig. 3*A*,*B*, black traces). We therefore classified these responses to two categories with reference to the solo odor: (1) t(B) responses different than the solo (Fig. 3*A*, t(B)≠solo) and (2) t(B) responses not significantly different than the solo (Fig. 3*A*, t(B)=solo, right). We first compared t(B) responses to the absolute level of the solo response (Fig. 3*A*, red vs black). The distribution of this comparison varied widely and was to a large extent odor specific (Fig. 3*C*). Pooling all odors together, 58% of responses were classified as being similar in both conditions (i.e., t(B)=solo; Fig. 3*E*, gray). Different fields from the same mice contributed similarly to the distribution of values between solo and t(B) (data not shown).

We also defined the relationship between the solo and t(B) responses differently, and considered the immediate background level preceding the stimulus as the baseline [Fig. 3*B*, blue vs black, referred to as t(B)-baseline]. Calculating the comparison this way shows again that the distribution of values was odor specific (Fig. 3*D*). Pooling all odors together using this method, 44% of responses were classified as being similar to the target in both conditions (i.e., t(B)-baseline=solo; Fig. 3*F*, gray). Comparing the two methods side by side showed a strong correlation between the two, strengthening the claim that target responses are odor specific (Fig. 3*G*). Note that in a subset of the odor pairs, the solo and target odors were not different in >50% of the cases in both measures [Fig. 3*C*,*D*, gray in Ea(Mp), Et(Ea), Mp(Ea), Ea(Et), Eb(Bu)]. Thus, in these odor pairs, the presence of background did not seem to affect the identity of most odors (see more below). The responses to these odors suggest that while the system suppresses background, it maintains the ability to identify an odor in the presence of background.

Given the heterogeneity of responses, one possible explanation for our results could be that the level of adaptation determines the level of similarity between an odor with background and without background. For example, a highly adapted response could “reset” the system to basal conditions, as if no background were present at all. To test this, we plotted the level of adaptation of each neuron versus how solo and t(B) compare. We found no obvious relationship between the level of adaptation and how similar the odor would be with or without a background (Fig. 3*H*). This result rules out a simple explanation whereby background suppression “resets” the system to detect an odor over background as new. Thus, residual activity in the OB during odorous background conditions does affect how new odors are processed.

### Mixture processing is different in the face of background

**Figure 4.**
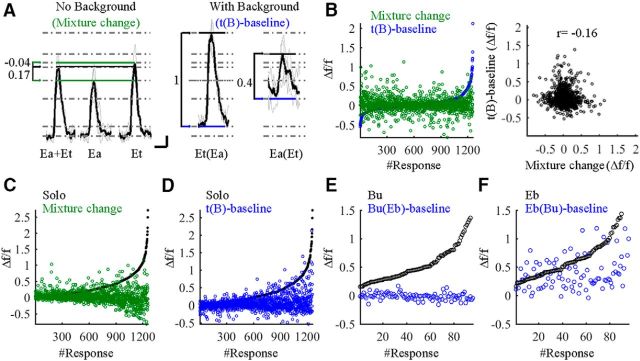
Mixture processing is modulated in the face of background. ***A***, Representative example of a MC, which was more sensitive to a target odor when background odor was present. The traces show the responses of this MC to five stimuli [Ea, Et, Ea+Et, Et(Ea), and Ea(Et)]. The gray dotted lines are references to baselines and response amplitudes denoting the changes in activation from one state to the next (arrows). For example, the transition from Ea to Ea+Et induces a 0.17 Δ*f*/*f* change in fluorescence (Mixture change; bottom green to black line). However, the response during background to the exact same stimulus was a change of 1 Δ*f*/*f* [t(B)-baseline; blue to black line in the Et(Ea) responses]. Similarly, the transition from Et to Ea+Et was a small decrease of −0.04 Δ*f*/*f* while a 0.4 Δ*f*/*f* increase was observed when background was present [Ea(Et)]. ***B***, Left, Scatter plots of t(B)-baseline for all odors sorted by their amplitude (blue) and the mixture change for the same odor combination in the same cell (green). Right, Plot of the correlations between mixture change (*x*-axis) and the change for the same mixture but when one odor is the background [t(B)-baseline, *y*-axis]. ***C***, Scatter plot of responses to the solo odor (black; sorted by amplitude) and the change in the mixture response by the same cell (green). Higher responses have lower mixture change (*y* = −0.3*x* + 0.3, *r* = −0.2). ***D***, Same as ***C*** but blue is the t(B)-baseline response. Here, higher responses do not show negative but rather more positive response changes (*y* = 0.5*x* + 0.3, *r* = 0.3). ***E***, ***F***, Same as in ***D*** but only for the odors Bu and Eb. In ***E***, Bu versus Bu(Eb) (*y* = −0.04*x* + 0.01, *r* = −0.26), and in ***F***, Eb versus Eb(Bu) (*y* = 0.36*x* + 0.17, *r* = 0.43). Note that background effects are not symmetric. See main text for details.

In our protocol, the stimulus during t(B) is the exact same stimulus as the binary mixture. The only difference between stimuli is the preceding background history. We thus compared how MCs responded to mixtures with regard to their responses to the mixture components with and without the history of background. To do so, we first analyzed the change MC responses undergo in the transition from a single odor into a mixture without background. This analysis is exemplified in one representative MC responding to an odor mixture (Ea+Et) as well as to the single odor components in isolation as solo odors (Ea and Et; Fig. 4*A*, No Background). The fluorescence change from the response to Ea into a mixture is evident as a stronger response to the mixture (Fig. 4*A*; quantified as “mixture change” = 0.17 Δ*f*/*f*). However, the change from the response to Et into the mixture is evident as a slightly weaker response to the mixture (Fig. 4*A*; mixture change = −0.04 Δ*f*/*f*). In both cases, the mixture response was smaller than the sum of the responses to the components. This phenomenon is known as mixture suppression, which is well documented in olfaction (Giraudet et al., 2002; Kadohisa and Wilson, 2006; Davison and Katz, 2007; Stettler and Axel, 2009; Shen et al., 2013).

We next tested whether mixture changes were themselves affected by background history. We used the analysis shown above in Figure 3*B*, which is the same as measuring the mixture change in the presence of background. In the example of Figure 4*A*, response changes to the mixture Et(Ea) were larger in the No Background condition compared with the With Background condition [Fig. 4*A*; compare Et(Ea)-baseline = 1 Δ*f*/*f*, mixture change = 0.17 Δ*f*/*f*]. Similarly, the change in Ea(Et) was larger than the No Background condition [compare Ea(Et)-baseline = 0.4 Δ*f*/*f*, mixture change = −0.04 Δ*f*/*f*]. Thus, the exact same chemical stimulus [i.e., Ea+Et, Et(Ea), Ea(Et)] induced different responses depending on the identity of the preceding stimulus history (i.e., no background, Ea as background, or Et as background). Further, a side-by-side comparison of response changes to mixtures with and without background showed no correlation (Fig. 4*B*), strengthening the claim for a history-dependence of MC responses.

To study more carefully the single-odor responses versus mixture responses under different background conditions, we looked for factors correlated with the mixture-suppressive behavior of MCs. In the No Background condition, stronger responses to an odor were negatively correlated with the resultant mixture-suppressive effect. Stronger responses to a single odor were accompanied by higher suppression in its mixture condition (Fig. 4*C*, black vs green; *y* = −0.3*x* + 0.3, *r* = −0.2). In contrast, when the exact same odors were preceded by background, the same neurons were no longer so mixture-suppressive (Fig. 4*D*; black vs blue; *y* = 0.5*x* + 0.3, *r* = 0.3). Notably, t(B) responses were not only odor specific but also background specific. The responses of MCs were different, depending whether an odor was background or target. In the most extreme odor pair, MCs responses showed almost no response to one odor as a target [Fig. 4*E*; Bu(Eb)] but remained sensitive to the other odor [Fig. 4*E*; Eb(Bu); Fig. 3*D*, compare gray dots in Eb(Bu) to Bu(Eb)]. Together, these data show that mixture processing by MCs strongly depends on the preceding history of the odor and its identity.

### Background adaptation and target responses over background are similar in the awake state and in lower odor concentrations

The results described thus far have been obtained by imaging MCs in anesthetized mice. Experimental conditions in anesthetized mice have some advantages over the awake state, such as being experimentally stable both mechanically and physiologically (Adam et al., 2014). Nevertheless, there are obviously physiological differences between the awake and anesthetized states. For example, in the OB of anesthetized mice, MCs were shown to have significantly stronger responses due to an attenuation of normal granule-cell inhibition (Rinberg et al., 2006; Kato et al., 2012; Cazakoff et al., 2014). Thus, we next carried out the exact same experiments in 4 of the 10 odor combinations in head-restrained awake mice.

**Figure 5.**
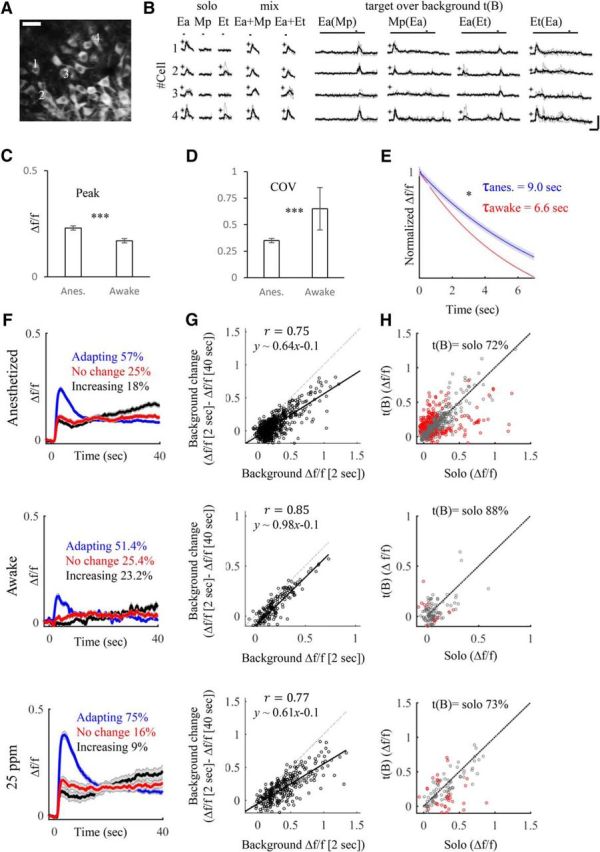
MC responses to t(B) in low-concentration and awake mice. ***A***, ***B***, Similar to Fig. 1*A*,*B* but in head-restrained awake mice. ***C***, Average of peak amplitude responses in awake and anesthetized mice. ***p = 9.6 × 10^−8^, *t* test. ***D***, Average of the coefficient-of-variance (COV) values in awake and anesthetized mice. ***p = 1.8 × 10^−8^, *t* test. ***E***, Average decay time from peak amplitudes to steady state in the awake (red) and anesthetized (blue) states, normalized to the peak. *p = 0.04, *t* test. Gray, SEM between different mice. ***F–H***, Side-by-side comparison of various response values using the same odor set to three different experimental conditions. Top, 250 ppm (anesthetized); middle, awake mice; bottom, 25 ppm (anesthetized). ***F***, Average traces of the adapting (blue), the stable (red), and the increasing (black) MC responses along a 40 s background stimulus. ***G***, Linear correlations between background intensity at the peak (peak at 2 s, *x*-axis) and after background change (peak at 2 s − peak at 40 s, *y*-axis). ***H***, Top, Scatter plot comparing the maximum calcium response of the target alone (solo; *x*-axis) to the target [t(B); *y*-axis]. Each data point is one cell–odor pair. Gray, Responses are not significantly different; red, responses are significantly different (unpaired 2-sample *t* test, *p* < 0.05). Middle, Same for awake mice. Bottom, 25 ppm concentration. On top of each graph is the percentage of gray dots from the total number of responses.

We implanted mice (*n* = 3) with a chronic window over the OB and imaged MCs 2 weeks after window implantation (Adam and Mizrahi, 2011; Adam et al., 2014). During imaging, mice were held in an awake, head-fixed position under the microscope as described previously (Kato et al., 2012). We measured calcium responses from MCs to the high odor concentration of 250 ppm (Fig. 5*A*,*B*). As reported before, in awake mice, calcium responses were weaker and fewer MCs were responsive. Of 186 imaged cells, only 43 were further analyzed (for a total of 112 cell–odor pairs).

Despite the weaker (Fig. 5*C*; peak amplitudes: anesthetized, 0.24 ± 0.01; awake, 0.17 ± 0.01, *p* = 1.8 × 10^−8^; *t* test), noisier (Fig. 5*D*; coefficient of variance: anesthetized, 0.35 ± 0.02; awake, 0.65 ± 0.2, *p* = 9.6 × 10^−8^; *t* test), and fewer responses of MCs in awake mice (responsive cells: anesthetized, 84%; awake, 64%), the results were qualitatively similar to the data collected from the anesthetized mice (one exception is a slightly faster average response in the awake state; Fig. 5*E*; response decay time: anesthetized, 9.0 s, *R*^2^ = 0.99; awake, 6.6 s, *R*^2^ = 0.82, *p* = 0.04; *t* test). On average, MCs adapted to ∼60% of their initial value of the background stimulus (anesthetized from 0.24 ± 0.01 at the peak to 0.14 ± 0.01 at 40 s; awake from 0.17 ± 0.01 to 0.11 ± 0.01 at 40 s). Single-MC analysis revealed that 51.4% of responses adapted, 25.4% remained similar, and 23.2% increased (Fig. 5*F*). Adaptation values were correlated strongly to the intensity of the background (Fig. 5*G*), and most (87.5%) responses to t(B) did not significantly differ when presented after 40 s background (Fig. 5*H*). Differences from the anesthetized state (e.g., fewer neurons passing significance from the diagonal) could also be due to the noisier nature of the weaker responses. Together, these data show that background adaptation and sensitivity to target odors over odorous backgrounds are qualitatively similar in awake and anesthetized mice.

To test the contribution of odor intensity to target sensitivity and adaptation, we repeated the same experiments using 10-fold lower odor concentration (25 ppm). As expected, fewer MCs were now responsive (59%), but odor-evoked responses had similar coefficient of variance values (0.31 ± 0.02) and decay dynamics (response decay time: 9.4 s, *R*^2^ = 0.99; similar to the 250 ppm, *p* = 0.36 but significantly different from the awake, *p* = 0.03; *t* test). Moreover, both background and t(B) responses were very similar (Fig. 5*F–H*; 25 ppm). We conclude that our results of both background adaptation and target responses remain consistent across physiological states and odor concentrations.

### Population coding is history dependent

**Figure 6.**
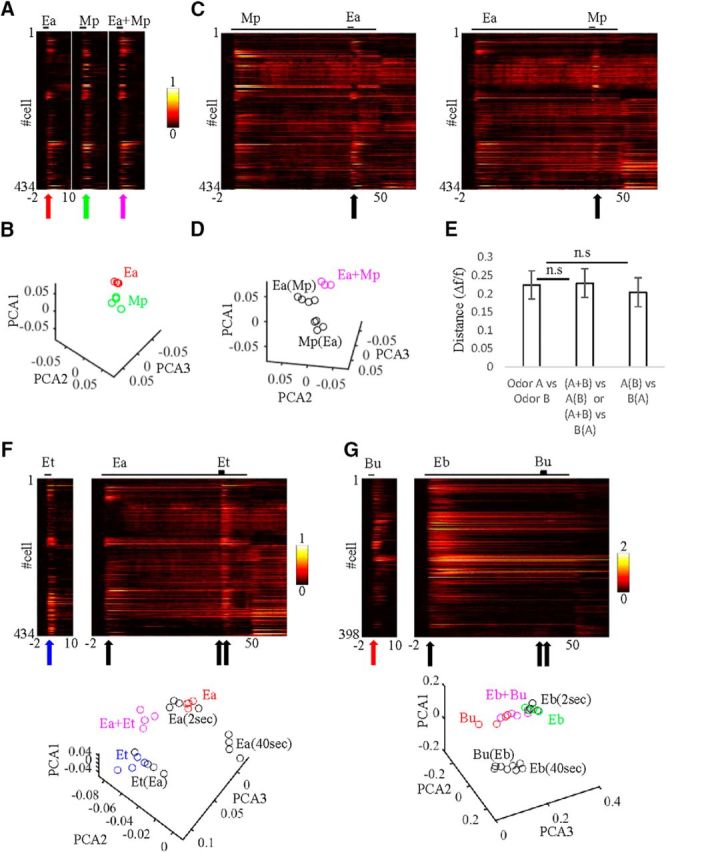
Population analysis of MC responses to single odors and mixtures in face of background. ***A***, Full response profiles of 434 imaged cells to single-odor stimuli (Ea, Mp), and their binary mixture (Ea+Mp). The stimulus is indicated above each plot by a black line. Color code is Δ*f*/*f*. Time 0 is the beginning of odor stimulus. Arrows are at *t* = 2 s and they correspond to the time in ***B***. ***B***, PCA plots of two odor-response vectors Ea (red) and Mp (green). Each dot represents a single trial from the time point indicated by the arrow in ***A*** (time, 2 s). ***C***, Full response profiles of all 434 imaged cells to Ea(Mp) and Mp(Ea) odor stimuli. Black arrow corresponds to ***D*** at *t* = 42 s. ***D***, PCA plots for the three mixture stimuli with different background histories. Ea+Mp, No background history (magenta); Ea(Mp), history of Mp as background (black); Mp(Ea), history of Ea as background (black). ***E***, Quantitative comparison of the Euclidean distances in PCA space for different pairwise comparisons where A and B represent different odors in a pair. Odor A versus Odor B, Distance between two different solo odor responses. A+B versus A(B) or A+B versus B(A), Distance between mixture with and without background (*p* = 0.95, Wilcoxon signed-rank test). A(B) versus B(A), Distance between mixtures with different backgrounds (*p* = 0.51, Wilcoxon signed-rank test). Error bar, SEM. ***F***, Top, Full response profiles of all 434 imaged cells to Et alone and Et(Ea) odor stimuli. Black and blue arrows correspond to the PCA plots at the bottom. Each circle in the PCA plot is an individual trial. After 2 s, Ea as background is adjacent in PCA space to Ea as solo. Later along the background (40 s), Ea differs in PCA space. At time 42, 2 s after target onset Et(Ea), responses are similar to Et in PCA space. ***G***, Same as ***F*** but for odors Bu(Eb). Response profiles from 398 cells.

Thus far, we only analyzed response properties of single neurons, which were responsive to a pair of the odors tested. Coding, however, is carried out by the collective activity of multiple neurons, many of which have variable and overlapping response profiles. For example, some neurons in our dataset responded to all three odors (Fig. 1*B*, cells 1–4), two odors (Fig. 1*F*, cell 2), one odor (Fig. 5*B*, cells 1, 3), or none (Fig. 1*F*, cell 3). Considering the whole population of neurons, we asked how does the population encode odors collectively with reference to recent background history. An example of all cells and all responses imaged from mice tested with the odor pair Ea–Mp are shown in Figure 6*A*,*C*.

As expected from the single-neuron responses, population responses to odors Ea and Mp in isolation were different (Fig. 6*A*; compare two left panels, red/green arrows). To evaluate how similar/different these population responses are, we used PCA. For odors Ea and Mp, PCA analysis shows distinct trajectories on the 2 s stimulus peak (Fig. 6*B*, red and green, respectively). Next, we found that the population response to an odor changed under different conditions of background history. For example, the population response of odor Ea on top of an Mp background [Ea(Mp)], was different than the response of odor Mp on top of an Ea background [Mp(Ea); Fig. 6*C*,*D*]. The difference is evident both in the raw traces (Fig. 6*C*, compare responses above black arrows) and in PCA space (Fig. 6*D*). Importantly, both conditions were different from the population response to the binary mixture Ea+Mp under the No Background condition (Fig. 6*D*, compare black, magenta).

To quantify the effects observed in the PCA analyses, we measured each population response as a multidimensional vector for each odor and calculated the Euclidean distances between the two vectors (see Materials and Methods). Specifically, we measured the distance in population responses between the mixture stimuli under different background conditions. For example, for the Ea and Mp odors, we looked at any pairwise mixture combination [Ea(Mp), Mp(Ea), Ea+Mp] versus the two different odors Ea versus Mp in isolation. On average, pooling all odors together, odor mixtures with different backgrounds showed distances of ∼0.2 Δ*f*/*f* and these were as distant as pairs of different odors in isolation (Fig. 6*E*, two left bars; *p* > 0.05, Wilcoxon rank-sum test). Importantly, almost all individual comparisons yielded similar distance values (with one exception for the odor pair Ia–Mp). Thus, the responses to the exact same odor mixture under different background conditions was as different as two different odors. We conclude that population responses to mixtures in the OB show strong history dependence.

### Impact of history-dependent responses

Next, we tested to what extent the MC population can detect an odor as such under continuous background activity. Since this quality was odor specific at the single-cell level (Fig. 3), we tested this by odor at the population level as well. When we limited our analysis only to those subsets of odors that showed stability in >50% of responses [Fig. 3*C*,*D*, Ea(Et), Et(Ea), Ea(Mp), Eb(Bu), Mp(Ea)], population responses to the target were similar to those to the isolated odor [Fig. 6*F*, compare Et, Et(Ea) in raw traces and in the PCA]. The distance between population response for a solo odor relative to its response as a target was shorter by ∼30% compared with the differences between different isolated odors (0.15 ± 0.01 Δ*f*/*f*). Notably, the ability of the system to maintain odor similarity in face of background was odor specific. Other odors [e.g., Bu vs Bu(Eb)] did not induce a target response at all in the face of the highly dominant Eb background (Fig. 6*G*). Similarly, other odor pairs like Mp(Pr) and Mp(Ia) were dominated by background, and showed a weak target response.

**Figure 7.**
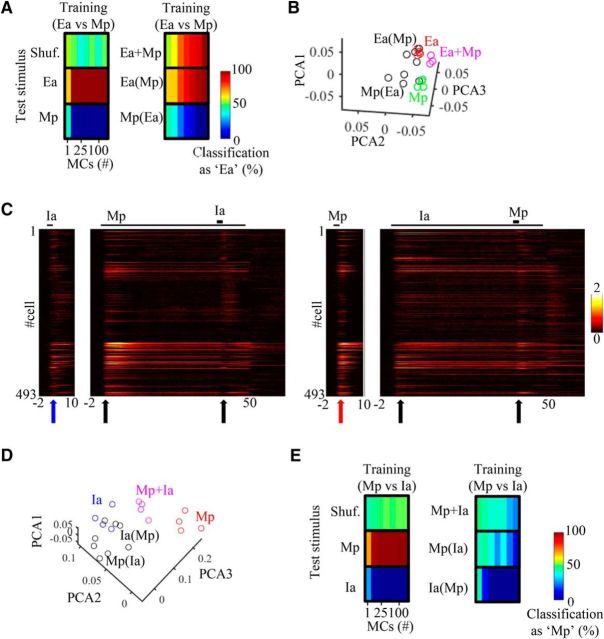
Odor classification on top of background. ***A***, Decision tree classification performance for different stimuli. The classifier trained on Ea versus Mp and tested on all five odor stimuli (as indicated on the left of each column). #MCs: 1, 2, 25, 50, 80, 100, 434. ***B***, PCA plots for odors Ea and Mp as solo odors, as targets, and as a mixture (same as in Fig. 6*F*,*G*). Note that Ea(Mp) is adjacent to Ea, and Mp(Ea) is adjacent to Mp. ***C***, Left, Full response profiles of all 493 imaged cells to Ia and Mp as solo odors, and under background conditions. ***D***, PCA plots of two odor-response vectors Mp (red) and Ia (blue). Each dot represents a single trial from the time point indicated by the arrows in ***C***. While Ia(Mp) is adjacent to Ia in PCA space, Mp is distant from Mp(Ia). ***E***, Same as in ***A*** but for the odors Mp and Ia. Classification of Mp(Ia) as Ia is due to the dominance of Ia as background.

Finally, we evaluated how a downstream decoder would classify MC responses to isolated odors and on top of background. We trained a classifier (decision tree; see Materials and Methods) on one pair of odors and then tested its classification decisions on different stimuli composed from these odors. Figure 7*A* shows results of the classifier after training MCs on the pair of odors Ea and Mp (Fig. 6*A*,*C*, raw traces). In each session, we randomly picked a number of MCs (from 1 to all imaged MCs, repeated 100 times) for training and testing. Classification accuracy for isolated odors was perfect for any population of >5 MCs (Fig. 7*A*, left). When the same MCs were tested on the mixture stimuli, which were not used for training, more MCs were needed for the decoder to reach clear classification (Fig. 7*A*, right). For this odor pair, the classifier correctly classified the target as such when the target was presented over background. For example, when Mp was the background and Ea(Mp) the target, the classifier identified the mixture as Ea [Fig. 7*A*, Ea(Mp)]. Similarly, when Ea was the background, and Mp(Ea) the target, it was now identified as Mp (Fig. 7*A*,*B*, PCA projections). This phenomenon was robust in all those odor combinations that remained sensitive to the target odor in >50% of their responses (Table 1). This result also remained similar in the low odor concentrations and in awake mice (Table 1). These results show that for most odor pairs, when an odor is presented over background, it is identified by the classifier as the target odor and not as the background.

**Table 1.** Response classification per field for odor response to Ea(Mp), Et(Ea), Mp(Ea), and Ea(Et) in awake and anesthetized mice

Test	Training		Test	Training	
	Ea	Mp		Ea	Et
Anesthetized 250 ppm					
Ea	100%	0%	Ea	100%	0%
Mp	0%	100%	Et	0%	100%
Ea + Mp	60%	40%	Ea + Et	80%	20%
Ea(Mp)	80%	20%	Ea(Et)	80%	20%
Mp(Ea)	20%	80%	Et(Ea)	60%	40%
Awake					
Ea	100%	0%	Ea	100%	0%
Mp	0%	100%	Et	0%	100%
Ea + Mp	50%	50%	Ea + Et	50%	50%
Ea(Mp)	75%	25%	Ea(Et)	25%	75%
Mp(Ea)	0%	100%	Et(Ea)	0%	100%
Anesthetized 25 ppm					
Ea	100%	0%	Ea	100%	0%
Mp	0%	100%	Et	0%	100%
Ea + Mp	33%	66%	Ea + Et	17%	83%
Ea(Mp)	50%	50%	Ea(Et)	66%	33%
Mp(Ea)	17%	83%	Et(Ea)	17%	83%

Fields with >25 MCs from all conditions were examined for stimulus classification (*n* = 5 anesthetized 250 ppm, 4 awake, and 6 anesthetized 25 ppm). Test stimuli are indicated in the left column. Values are the average percentages of fields classified as one of the training stimuli.

As mentioned above, some target odors induced rather weak responses when presented over background [Fig. 3*D*, Bu(Eb), Mp(Ia), Mp(Pr)]. In those odors, background dominated the responsivity of the network, masking additional responses [Fig. 7*C*,*D*, compare Mp, Mp(Ia)]. In these cases, a decoder trained on the single odors often made mistakes in identifying the t(B) mixtures as the correct target odor [Fig. 7*E*; see decoder performance on Mp(Ia)]. For those dominant background odors, further processing beyond the OB would be needed to change background activity to a level that will allow a reliable detection of a target. Alternatively, these background odors could be difficult to detect perceptually under these conditions.

## Discussion

We imaged odor-evoked activity of MCs to continuous stimuli as well as to t(B). The vast majority of MCs changed their activity levels during the prolonged odor presentation, suggesting that odors are dynamically encoded in the OB at slow time scales. Despite some heterogeneity, most MCs showed adaption levels scaled by stimulus intensity. When a target odor was presented over an odorous background, MC-response patterns were complex across all odors. Specific responses to t(B) varied based on the identity of the odor but were always affected by the presence of background. This suggests that odor processing is dynamically updated in a history-dependent context as early as the OB.

### Background suppression and target sensitivity

The OB is not a simple relay station. Olfactory sensory neurons (OSNs) send odor input into the OB, which is then processed by several local inhibitory and excitatory networks (Wilson and Mainen, 2006; Murthy, 2011). Odor input is also modulated by centrifugal input or feedback connections projecting back into the OB (Shea et al., 2008; Petzold et al., 2009; Boyd et al., 2012; Ma and Luo, 2012; Markopoulos et al., 2012). As a result, MC output is a transformed version of the initial odor input (Kikuta et al., 2013; Adam et al., 2014). Accumulating evidence focusing mainly on short time-scale responses suggest that MC output may reflect numerous computations, such as those related to pattern decorrelation (Padmanabhan and Urban, 2010; Friedrich and Wiechert, 2014), contrast enhancement (Cleland, 2010), and normalization (Cleland et al., 2007; Olsen et al., 2010), and even “higher” computations, such as those that reflect odor value (Doucette and Restrepo, 2008) or animal state (Pírez and Wachowiak, 2008). We present a description of single-cell and population response profiles of MCs with regard to how they process odor input at slow time scales.

The responses we observed in MCs are potentially a result of upstream, local, or downstream computations. The initial candidates for shaping MC adaptive responses are the OSNs, located upstream in the hierarchy. Indeed, the initial phase of background suppression most likely carries a strong OSN component, which show adaptation both *in vitro* and *in vivo* (Zufall and Leinders-Zufall, 2000; Bradley et al., 2005; Kleene, 2008; De Palo et al., 2013). For example, Lecoq et al. (2009) studied adaptation to 4 s stimuli and found that the adaptation in glutamatergic axons of OSNs can explain glomerular adaptation as measured by local field potentials, suggesting that the OSN–MC synapse is the sole locus responsible for this adaptation. Our measurements were different than those of the Lecoq et al. study both in the duration of the stimulus (here, 40 s) and in the location of measurement (i.e., MC somata). Thus, while the initial adaptive phase in the glomerulus is most likely explained by OSN adaptation, the developing responses in MC somata along the 40 s stimulus would involve other downstream synapses, which are most certainly active. In the fly, Carafo (2016) made direct measurements of adaptation from projections neurons (PNs; the fly analog of MCs) alongside their cognate OSNs. He found that PN adaption is greater than OSN adaptation, and described additional sources for adaptation downstream of OSNs. Notably, the responses of MCs that we measured were not all adaptive; 28% of neurons did not adapt and significant portions of these increased their responses. The anatomical complexity of OB circuits downstream of the OSN–MC synapse is thought to be more complex in the mouse than in the fly, and remain likely candidates to contribute to MC-response profiles. Additionally, OSN responses to mixtures are often reported as cumulative (but see Duchamp-Viret et al., 2003; Lin et al., 2005; Verhagen et al., 2007; Rospars et al., 2008; Fletcher, 2011; Su et al., 2011; Saha et al., 2013), while MC responses to mixtures are suppressive (Fig. 4; Giraudet et al., 2002; Davison and Katz, 2007). Thus, mixture stimuli most likely recruit additional mechanisms (e.g., inhibitory circuits) to suppress MC activity to mixtures. Future studies should produce a more mechanistic explanation of how the slow responses from different circuit components develop in the OB.

There are several other candidate subpopulations that provide inhibitory (and excitatory) input to MCs and could further contribute to the effects we observe. For example, both intraglomerular and interglomerular inhibition is known to shape the amplitude and temporal activity of odor responses (Shao et al., 2012; Kato et al., 2013; Miyamichi et al., 2013; Banerjee et al., 2015). These could potentially contribute to the suppression of continuous background odors in an odor-specific manner. Granule cells are also locally active and could contribute to achieve specificity (Arevian et al., 2008; Koulakov and Rinberg, 2011; Kollo et al., 2014). Given its slow time to evolve, sources of adaptation are not necessarily fast or direct. Thus, downstream sources, such as cortical feedback, are well positioned to play a role in MC adaptation (Boyd et al., 2012; Markopoulos et al., 2012). Recently, feedback projections were shown to mediate odor-specific effects on MCs, a property that could serve as a mechanism for odor-specific MC adaptation (Boyd et al., 2015; Otazu et al., 2015). Adaptation could involve numerous inhibitory sources that operate simultaneously and or consecutively. For example, granule cells may be activated by centrifugal feedback and indirectly suppress MCs (Koulakov and Rinberg, 2011). In fact, centrifugal feedback as a mechanism for adaptation was proposed 25 years ago by modeling studies (Li, 1990), but with little empirical support.

### History-dependent processing: behavior implications

In nature, odor stimuli are composed of complex mixtures. However, here we used pure odorants that are neither behaviorally meaningful nor complex. Behavioral correlates of odor sensitivity over background using simple mixtures have been described. For example, Linster and colleagues trained rats to associate a simple odor with water reward (2007). Rats were then adapted to a prolonged constant background odor resulting in a decrease of their behavioral response to the background. Rats remained sensitive to new odors presented on top of the adapted background, showing that adaptation, even to simple mixtures, affects perception. Activities of PNs in the locust antennal lobe (analogous to the OB in insects) have also been found to correlate with perception of target odors over background in an odor-dependent manner (Saha et al., 2013). MC activity has been shown to be modulated by experience (Kay and Laurent, 1999; Mandairon and Linster, 2009) and to encode odor value (Doucette and Restrepo, 2008; Restrepo et al., 2009; Doucette et al., 2011). We speculate that behaviorally meaningful odors would have higher sensitivity in face of background. Exploring whether naturally meaningful (aversive or attractive) odors or learned odors share similar properties to simple odors could shed light on the mechanisms that support these computations in the OB.

The rich behavioral repertoire of mice, like social recognition or their ability to locate foods and avoid predators in odor-rich environments, must rely heavily on segregating salient odors from background. In real life, where mixtures are complex, we expect even stronger adaptation as more inhibitory channels are activated. Identifying neural correlates of background suppression and history-dependent processing so early in the olfactory hierarchy sets a substrate for higher computations performed in cortical regions like, for example, those computations needed for object recognition in complex odor scenes.
